# Occupancy winners in tropical protected forests: a pantropical analysis

**DOI:** 10.1098/rspb.2022.0457

**Published:** 2022-07-13

**Authors:** Asunción Semper-Pascual, Richard Bischof, Cyril Milleret, Lydia Beaudrot, Andrea F. Vallejo-Vargas, Jorge A. Ahumada, Emmanuel Akampurira, Robert Bitariho, Santiago Espinosa, Patrick A. Jansen, Cisquet Kiebou-Opepa, Marcela Guimarães Moreira Lima, Emanuel H. Martin, Badru Mugerwa, Francesco Rovero, Julia Salvador, Fernanda Santos, Eustrate Uzabaho, Douglas Sheil

**Affiliations:** ^1^ Faculty of Environmental Sciences and Natural Resource Management, Norwegian University of Life Sciences, Ås, Norway; ^2^ Program in Ecology and Evolutionary Biology, Department of BioSciences, Rice University, Houston, USA; ^3^ Moore Center for Science, Conservation International, Arlington, VA, USA; ^4^ Institute of Tropical Forest Conservation, Mbarara University of Science and Technology, Kabale, Uganda; ^5^ Conflict Research Group, Ghent University, Belgium; ^6^ Facultad de Ciencias, Universidad Autónoma de San Luis Potosí, San Luis Potosí, Mexico; ^7^ Escuela de Biología, Pontificia Universidad Católica del Ecuador, Quito, Ecuador; ^8^ Smithsonian Tropical Research Institute, Panama City, Panama; ^9^ Wildlife Ecology and Conservation Group, Wageningen University, Wageningen, The Netherlands; ^10^ Wildlife Conservation Society - Congo Program, Brazzaville, Republic of the Congo; ^11^ Nouabalé-Ndoki Foundation, Brazzaville, Republic of the Congo; ^12^ Biogeography of Conservation and Macroecology Laboratory, Institute of Biological Sciences, Universidade Federal do Pará, Pará, Brazil; ^13^ Department of Wildlife Management, College of African Wildlife Management, Mweka, Moshi, Tanzania; ^14^ Department of Ecological Dynamics, Leibniz Institute for Zoo and Wildlife Research, Berlin, Germany; ^15^ Department of Ecology, Technische Universität Berlin, Berlin, Germany; ^16^ Department of Biology, University of Florence, Florence, Italy; ^17^ MUSE-Museo delle Scienze, Trento, Italy; ^18^ Wildlife Conservation Society, Quito, Ecuador; ^19^ Museu Paraense Emílio Goeldi, Belém, Pará, Brazil; ^20^ International Gorilla Conservation Programme, Musanze, Rwanda; ^21^ Forest Ecology and Forest Management Group, Wageningen University and Research, Wageningen, The Netherlands; ^22^ Center for International Forestry Research, Bogor, Indonesia

**Keywords:** biodiversity patterns, camera-traps, community structure, functional traits, habitat specialization, hierarchical occupancy modelling

## Abstract

The structure of forest mammal communities appears surprisingly consistent across the continental tropics, presumably due to convergent evolution in similar environments. Whether such consistency extends to mammal occupancy, despite variation in species characteristics and context, remains unclear. Here we ask whether we can predict occupancy patterns and, if so, whether these relationships are consistent across biogeographic regions. Specifically, we assessed how mammal feeding guild, body mass and ecological specialization relate to occupancy in protected forests across the tropics. We used standardized camera-trap data (1002 camera-trap locations and 2–10 years of data) and a hierarchical Bayesian occupancy model. We found that occupancy varied by regions, and certain species characteristics explained much of this variation. Herbivores consistently had the highest occupancy. However, only in the Neotropics did we detect a significant effect of body mass on occupancy: large mammals had lowest occupancy. Importantly, habitat specialists generally had higher occupancy than generalists, though this was reversed in the Indo-Malayan sites. We conclude that habitat specialization is key for understanding variation in mammal occupancy across regions, and that habitat specialists often benefit more from protected areas, than do generalists. The contrasting examples seen in the Indo-Malayan region probably reflect distinct anthropogenic pressures.

## 1. Introduction

Although tropical forests cover less than 7% of Earth's land surface, they support most of the world's biodiversity [1]. The largest remaining tracts of tropical forest are located in South America, Central Africa and Southeast Asia [2]. Despite the superficial similarities of these habitats, the taxonomic composition of their communities vary across regions [3]. However, little is known about the variation in the functional structure of mammal communities between tropical forests [4]. Mammals are well represented taxonomically and play important roles in these systems [5], but until recently, they have remained difficult to survey in a robust and consistent manner due to their mobility and elusive behaviours.

To date, most studies assessing large-scale biodiversity patterns in mammals have focused on species identities rather than their characteristics, such as body mass and feeding guild [6]. Such characteristics, known as ‘functional traits’, determine the ecological relationship between a species and its environment [6]. The few studies that have assessed spatial patterns of such functional traits in tropical regions concluded that the functional composition of tropical forest mammals is largely consistent among regions, likely due to the age and stability of these forests, as well as similarities in climate and day length [4,7]. These studies, used species richness to determine functional composition and diversity [8,9] rather than ‘occupancy’—defined as the proportion of sites occupied by a species [10].

Occupancy is a basic quantity used to monitor wildlife populations and can be interpreted both as an indicator of abundance [11,12] and distribution [13]. Additionally, occupancy models enable the estimation of detection probability and thus account for imperfect detection [10]. This is important because individuals can remain undetected while they are present, and variation in detectability across species and landscapes can lead to flawed inferences about occupancy [10,14]. Accounting for imperfect detection is useful in tropical forests, where many species are rare or elusive [15]. Yet, most large-scale tropical studies focusing on functional traits have relied on map-based data or species lists, which can affect the ecological patterns found (e.g. [16,17]). For example, range maps often contain errors of commission (i.e. species are thought to be present in locations where they are absent [18,19]). Furthermore, species lists are inadequate to model detection probability and neglect differences in sampling effort [20,21]. Standardized *in situ* camera-trap data collected across the global tropics provides a previously unavailable data source to examine how ecological and evolutionary processes might affect occupancy of tropical mammal communities. In any case, recent research indicates that the relationship between mammal functional dispersion (i.e. spread of species traits in a community) and primary productivity in tropical forests can be better understood by accounting for species' occupancies [9].

Investigating the relationship between occupancy and species characteristics such as feeding guild or body mass provides insight into how functional groups are distributed [9,22]. For example, biomass decreases with increasing trophic level, and abundance or occupancy is therefore expected to be higher for herbivores than for carnivores [5,23]. However, herbivores occupancy can vary across space, as plant resources are not equally available across tropical regions [24] and there is spatial variation in top-down control from predators and humans [5,25]. Regarding the effect of body mass on mammal occupancy, larger mammals may have lower occupancy than smaller species. This reflects three factors: their low reproductive rates which may slow down their response to environmental and anthropogenic change [26]; their high metabolic rates, and thus high energy demands which makes them more vulnerable to food scarcity [27]; and their vulnerability to hunting [28].

The importance of other species characteristics such as the degree of ecological specialization has been largely neglected when assessing global biodiversity patterns. Nevertheless, understanding how the degree of ecological specialization correlates with occupancy is critically important as specialist species (e.g. species that occupy a low number of habitat types or a limited geographical range) are typically at increased extinction risk when compared to generalists [29,30]. In fact, previous research suggests that specialist species are being replaced by generalist species as a result of global change, altering species communities and ecosystem functioning [30]. In protected areas where anthropogenic disturbance is expected to be minimal, occupancy of habitat specialists may nonetheless be higher than that of habitat generalists which are more suited to modified and heterogeneous habitats [31]. A study on tropical birds concluded that protected areas do not contain a higher total number of species than unprotected areas but retain a higher number of specialist species [32]. Whether the occupancy of specialists is higher than the occupancy of generalists in protected areas is largely unknown. Yet, answering this question is crucial, as establishment of protected areas is a key conservation strategy [33], especially for species judged vulnerable to loss which are often habitat specialists (electronic supplementary material, figure S1) [32]. Assessing how species characteristics including habitat specialization relate to mammal occupancy can help clarify how effective protected areas are for different groups of species.

Here, we assessed occupancy patterns of terrestrial mammals across protected areas from three tropical regions—Neotropics, Afrotropics and Indo-Malayan—and quantified differences in occupancy patterns among regions. We used camera-trap data from an extensive standardized tropical forest monitoring system [22,34], in combination with occupancy models. Specifically, we asked:

(1) How consistent are the relationships between mammal occupancy and species charactersitics among biogeographic regions?
(2) How do body mass and feeding guild relate to mammal occupancy?
(3) What is the relationship between species occupancy and their degree of habitat specialization?

We predicted that occupancy patterns would be consistent across tropical regions due to the age and stability of tropical forests. We also predicted that herbivores would have the highest occupancy. This is because within mammals, herbivores are the most basal trophic level and both energy and biomass decrease from lower to higher trophic levels. Large species (independently of trophic guild) would have lower occupancy than medium-size species as body mass is often positively correlated with vulnerability (e.g. reproductive rates decrease and hunting pressure increases with body mass). Finally, although specialists are more sensitive to global change than generalists, we predicted that habitat specialists would have higher occupancy because we focused on protected areas, where habitat degradation and anthropogenic disturbance is expected to be low (or managed).

## 2. Methods

### (a) Field sites and camera-trap data

**Figure 1.**
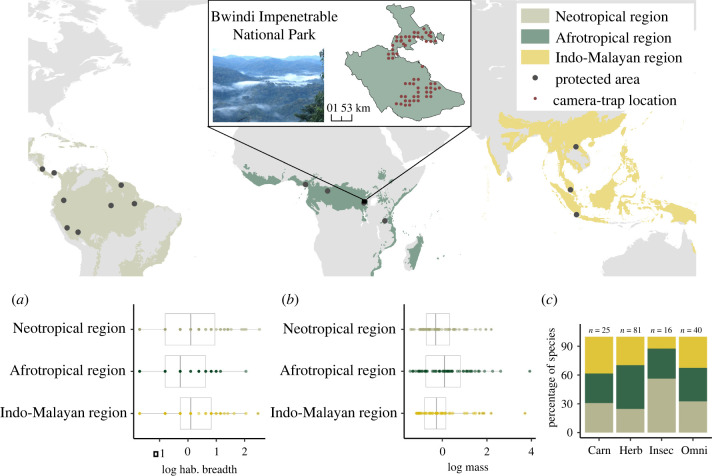
Distribution of tropical and subtropical rain forests in the Neotropical, Afrotropical and Indo-Malayan region including TEAM protected areas (main map). Inset map shows camera-trap locations in one protected area (Bwindi Impenetrable National Park). Bottom panels show the distribution of values of the species-specific covariates for each biogeographic region: (*a*) body mass, (*b*) habitat breadth and (*c*) the proportion of each feeding guild per biogeographic region (*c*): carnivores (Carn), herbivores (Herb), insectivores (Insec) and omnivores (Omni). Numbers on top of the bars in (*c*) indicate the total number of species in each feeding guild. Body mass and habitat breadth values are log-transformed and standardized. (Online version in colour.)

We used data from the Tropical Ecology Assessment and Monitoring (TEAM) Network, a standardized tropical forest camera-trap monitoring system [22,34]. The TEAM data comprises camera-trap data from protected areas located across three different biogeographic regions (Neotropical, Afrotropical and Indo-Malayan) (figure 1). In each area, camera-traps are deployed at 60–90 locations at a density of 1 camera per 1–2 km^2^ (figure 1 and electronic supplementary material, figure S2). Each camera-trap is deployed for a minimum of 30 days during the dry season (i.e. months with less than 100 mm average rainfall or the driest part of the year in the absence of dry season), although cameras may be active for less than 30 days due to damage or failure. Further details on the field methods are provided in electronic supplementary material, appendix S1.

We analysed data from 16 protected areas collected between 2008 and 2017 (8 protected areas in the Neotropics, 5 in the Afrotropics and 3 in the Indo-Malayan region), where each protected area was surveyed for a minimum of 2 years and a maximum of 10 years (mean = 6.44 years) (electronic supplementary material, table S1). We excluded camera-trap locations for which inconsistencies in the date-time stamps were found, thus yielding a sample size of 1002 camera-trap locations, with the number of sites per protected area ranging from 60 to 89 (mean = 62.63 sites) (electronic supplementary material, table S1). Moreover, we removed observations of terrestrial mammal species with an average body mass less than 1 kg, as smaller species can be difficult to detect and identify when using camera-traps [35].

### (b) Multi-species occupancy modelling

We used a hierarchical Bayesian multi-species single-season occupancy model [36] to assess how mammal occupancy differed between biogeographic regions, and how these differences related to species characteristics. Multi-species occupancy models are an extension of the single-species occupancy model [10] where the parameters of each species are treated as random effects, and species-specific effects are drawn from a common distribution (community-level distribution) [36]. This modelling framework typically improves occupancy estimates (i.e. reduce prediction error or uncertainty intervals) [37]. We further extended the multi-species occupancy model to use all data collected across multiple protected areas and years. Thus, species-specific random effects were drawn from a bioregion-level distribution (data from all communities belonging to the same biogeographic region). Definition of the random effect is indicated below. A dynamic occupancy model was judged unnecessary because we sought to assess how species characteristics relate to occupancy rather than to local colonization and extinction.

Occupancy models use data from sampling sites visited on multiple occasions within a period assumed to be closed to changes in occupancy [10]. We defined a sampling occasion as seven consecutive camera-trap days [38,39] and assumed that camera-trap locations were closed to changes in occupancy for a maximum of four sampling occasions (i.e. we included data from the first 28 camera-trap days). Inclusion of a given species for a given protected area was conditional on that species having been detected at least once by the TEAM camera-traps. Consequently, we did not use data augmentation, as is common in multi-species occupancy studies that aim to estimate the true number of species present in a community [40].

Our hierarchical model consisted of two sub-models: a sub-model for the ecological process in which the true occurrence of species *k* at site *i*, year *t*, protected area *a* and biogeographic region *b* (*z_itkab_*) is an unobserved latent variable represented by a Bernoulli process (*z_itkab_* ∼ Bernoulli [*Ψ_tkab_*], where *Ψ_tkab_* represents the occupancy probability), and a sub-model for the observation process, in which the detection of species *k* for occasion *j* at site *i*, year *t*, protected area *a* and bioregion *b* is represented by a Bernoulli process (y*_ijtkab_* ∼ Bernoulli [*z_itkab_* × *p_tkab_*], where *p_tkab_* represents the detection probability and is conditional on the site being occupied, i.e. *z_itkab_* = 1).

### (c) Covariates on detection

We modelled detection probability as a linear function of species-specific covariates known to correlate with detection of animals by camera-traps [41]:

$$\mathrm{logit}(\;p_{tkab})=\;\alpha_{\;p,tkab}+{\beta_{p}}_{1_{b}}\times \mathrm{Forest}\;{\mathrm{strata}}_{k}+\;{\beta_{p}}_{2_{b}}\times \mathrm{Body}\;{\mathrm{mass}}_{k}+\;{\beta_{p}}_{3_{b}}\times \mathrm{Forest}\;{\mathrm{strata}}_{k}\times \mathrm{Body}\;{\mathrm{mass}}_{k},$$

where $\alpha_{p}$ is the species-specific random effects drawn from a shared bioregion-level distribution and is defined as as *α*_p,*tkab*_ ∼ *Normal(µ_b_, σ^2^_b_)*, with *µ_b_* representing the mean parameter value of all the species belonging to the same biogeographic region, and *σ^2^_b_* the variance around that mean. By drawing one intercept per year (*α*_p,*tkab*_) we accounted for differences in occupancy over time. *β_p_* are the coefficient describing, for each biogeographic region, the relationship between detection probability and the following covariates (and their interaction):

— *Body mass*, defined as average adult body mass, reflects the amount and quality of resources that a species requires to survive, as well as home range size, fecundity or susceptibility to predation.
— *Forest strata* represents the foraging stratum: ground-dwelling and arboreal/scansorial species. Ground-dwelling species serve as the reference group and in the model is represented by the intercept.

### (d) Covariates on occupancy

We modelled occupancy probability as a function of both species- and protected areas-specific covariates:

$$\begin{array}{rl} \mathrm{logit}(\Psi_{tkab}) & =\alpha_{\Psi ,tkab}+\beta_{\Psi 1_{b}}\times \mathrm{Body}\;{\mathrm{mass}}_{k}+\;\beta_{\Psi 2_{b}}\times \mathrm{Feeding}\;{\mathrm{guild}}_{k}\\ & \;+\beta_{\Psi 3_{b}}\times \mathrm{Habitat}\;{\mathrm{breadth}}_{k};+\beta_{\Psi 4_{b}}\\ & \;\times \mathrm{Division}\;{\mathrm{index}}_{a}+\;\beta_{\Psi 5_{b}}\times \mathrm{Human}\;{\mathrm{population}}_{a}, \end{array}$$

where *α_ψ_* is the species-specific random effects drawn from a shared bioregion-level distribution, similarly to the detection probability described above. The coefficients (*β_ψ_*) describe, for each biogeographic region, the relationship between occupancy probability and the following covariates:

— *Body mass* (defined above).
— *Feeding guild* reflects the type of dietary resources needed, but also potential interactions with other species (e.g. competition or predation). We defined carnivores as species feeding on greater than or equal to 80% vertebrates, herbivores species feeding on greater than or equal to 80% plant materials, insectivores species feeding on greater than or equal to 80% insects, and omnivores the rest of species. Herbivores serve as the reference group and in the model and is represented by the intercept.
— *Habitat breadth* represents the degree of ecological specialization and is measured as the number of IUCN habitat types occupied by a species.

Even though anthropogenic influence is minimal inside protected areas, we accounted for differences in environmental conditions and anthropogenic threats in the surroundings. Therefore, around each camera-trap array within a protected area, we created a 10 km buffer which included protected and unprotected area. We calculated two landscape-scale covariates in the buffer:

— *Division index* represents forest fragmentation [42].
— *Human population* reflects human disturbances.

Because camera-trap arrays were spaced by more than 10 km in some protected areas (see electronic supplementary material, figure S2), we calculated the landscape-scale covariates for each of the camera-trap arrays located within a given protected area. We selected the buffer size based on sensitivity analyses (electronic supplementary material, figure S3).

We additionally calculated *percentage of forest* as a measure of habitat availability, but we did not include it in our model as it was correlated with *division index* (*r* > 0.70). Further details on the calculation of the spatial covariates are provided in electronic supplementary material, appendix S2 and figure S4.

We used species characteristics from different published databases for *body mass* and *feeding guild* [43], for *forest strata* [44] for *habitat breadth* [45]. A complete list of the mammal species included in this study with their functional information is provided in electronic supplementary material, table S2 and figure S5. For the spatial covariates, we used forest cover data [46] to calculate the *division index*, and global human settlement data [47] to extract *human population*. For the analysis, we log-transformed *body mass*, *habitat breadth* and *human population* (before standardization).

### (e) Model fitting

We fitted our model using Markov chain Monte Carlo (MCMC) methods via the package *nimble* in R [48,49]. We used uninformative or weakly informative priors (electronic supplementary material, table S3) and ran 6 chains of 320 000 MCMC iterations each, discarding the first 40 000 iterations as burn-in. We assessed MCMC convergence and mixing by visually inspecting trace plots and by calculating the Gelman–Rubin statistic for each parameter of interest, where values lower than 1.1 indicated convergence [50]. Model code is provided in electronic supplementary material, appendix S2.

We used the mean of the posterior distribution and the associated 95% Bayesian credible intervals (95% CI) of each beta coefficient to assess the effect of covariates on detection and occupancy. In our model, we estimated one beta coefficient per region, meaning that the relationships between occupancy and covariates were reported at the biogeographic region level. Variation in the number of protected areas, camera-trap locations or years among biogeographic regions did not affect occupancy estimates, but the uncertainty around those estimates (i.e. wider or narrower 95% CI).

## 3. Results

The sampling included 154 406 camera-trap days (considering all protected areas and years of data). Camera-traps detected 162 terrestrial mammal species in total (body mass greater than 1 kg), ranging from 12 to 34 (median = 24.5) per protected area (electronic supplementary material, table S1). Body mass of the species detected ranged from 1.06 to 4400 kg (median = 7.14) and habitat breadth from 1 to 26 habitat types (median = 4) (figure 1; electronic supplementary material, table S2). The species included 81 herbivores, 40 omnivores, 25 carnivores and 16 insectivores (figure 1), and forest strata groups included 106 ground-dwelling species and 56 arboreal/scansorial species.

**Figure 2.**
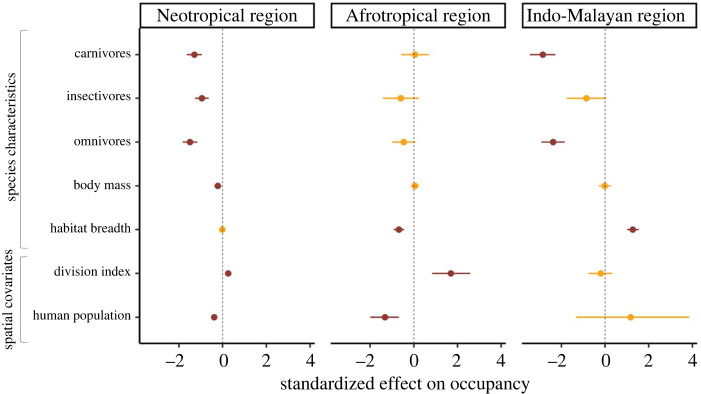
Standardized beta occupancy coefficients and 95% Bayesian credible intervals (95% CI) for all covariates and the three biogeographic regions. The effect of a covariate on occupancy was considered to be significant (red bars) when the 95% CI did not overlap zero (dashed vertical lines). (Online version in colour.)

**Figure 3.**
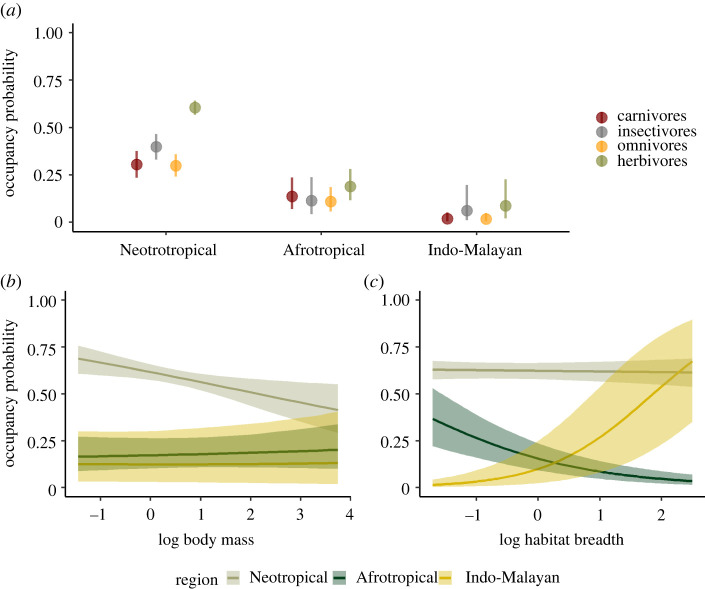
Occupancy probability of feeding guilds (carnivores, insectivores, omnivores and herbivores) across biogeographic regions (*a*). Occupancy probability in relation to body mass (*b*) and habitat breadth (*c*) for herbivores (reference value) across biogeographic regions. Plots show the mean predicted occupancy probability given the average body mass and habitat breadth observed in each biogeographic region, and 95% Bayesian credible intervals. Body mass and habitat breadth in panel *b* and *c* are log-transformed and standardized. (Online version in colour.)

Mammal occupancy varied across regions—highest in the Neotropics and lowest in the Indo-Malayan region—and was related to feeding guild, body mass and habitat specialization. The form and magnitude of these relationships differed among regions (figures 2 and 3). Among feeding guilds, herbivores had the highest occupancy in all three regions (figure 3). By contrast, omnivores and carnivores tended to have the lowest occupancy, whereas insectivores generally had intermediate values. The highest occupancy values were achieved by Neotropical herbivores (mean occupancy = 0.60; 95% CI = 0.56, 0.64) (figure 3). The Indo-Malayan region had the lowest values, particularly for carnivores (mean occupancy = 0.02; 95% CI = 0.00, 0.05) and omnivores (mean occupancy = 0.02; 95% CI = 0.00, 0.05) (figure 3). The relationship of body mass on occupancy was only statistically significant in the Neotropics, where occupancy decreased with increasing body mass (*β* = −0.22; 95% CI = −0.38, −0.06) (figures 2 and 3).

The habitat breadth–occupancy relationship varied among regions (figure 2). In the Afrotropics, occupancy decreased with habitat breadth (*β* = −0.68; 95% CI = −0.92, −0.45), meaning that habitat specialists had higher occupancy than generalists. In the Indo-Malayan region, however, this relationship was positive (*β* = 1.27; 95% CI = 1.01, 1.55), meaning that here habitat generalists had higher occupancy than specialists. Finally, habitat breadth was not significantly related to mammal occupancy in the Neotropics (figure 2).

Regarding landscape covariates, occupancy was negatively related to human population in the Neotropics (*β* = −0.39; 95% CI = −0.53, −0.24) and Afrotropics (*β* = −1.32; 95% CI = −1.99, −0.68), while division index was positively related to occupancy (Neotropics: *β* = 0.25; 95% CI = 0.12, 0.39; Afrotropics: *β* = 1.69; 95% CI = 0.84, 2.58) (figure 2). Neither covariate was significant in the Indo-Malayan region (figure 2).

Finally, detection probabilities varied by forest strata and body mass. Ground-dwelling species had a higher detection probability than species typically considered arboreal and scansorial (electronic supplementary material, figure S7 and S8). Additionally, detection decreased with body mass, except for arboreal and scansorial species from the Afrotropical and Indo-Malayan region, for which detection increased with increasing body mass (electronic supplementary material, figure S8).

## 4. Discussion

Tropical forests harbour hundreds of mammal species, but how their occupancy patterns vary remains largely unknown. Focusing on protected areas where anthropogenic disturbance is minimal, we quantified occupancy of medium-to-large terrestrial mammals across three tropical forest regions. We found that while some occupancy patterns were comparable among regions, others were region-specific. Herbivores had the highest occupancy in all regions. A relationship with body mass was only significant in the Neotropics, where large species had lower occupancy than medium-size species. Interestingly, the contrasting occupancy patterns we observed among regions showed a distinct relationship with habitat specialization: while specialists had highest occupancy in the Afrotropics, the opposite occurred in the Indo-Malayan region. Overall, our study indicates how mammal occupancy varies among regions, and how species characteristics such as feeding guild, body mass and habitat specialization may help characterize this variation. Such differences presumably reflect the past and present biogeographic characteristics of each site and region.

### (a) Herbivores and large species have the highest occupancy

To our knowledge, our study is the first to estimate and compare occupancy of different feeding guilds across biogeographic regions (but see [22] who compared occupancy among seven tropical protected areas). Herbivores showed the highest occupancy. Viewed as a measure of abundance, this is expected because herbivores are at the bottom of mammals’ trophic levels [5,51]. Occupancy of herbivores was notably higher in the Neotropics. This region holds more plant species than the other regions [24], potentially explaining the high occupancy of herbivores. Alternative explanations for this finding include a possible low hunting pressures in the Neotropics in comparison to the Afrotropical and Indo-Malayan regions [52,53], and/or the low abundance of large carnivores [3,54–56]. Indeed, studies suggest that the increase in population densities of herbivore species in one of the Neotropical protected area included in this study (Barro Colorado Island, Panama) is associated with the absence of large predators such as jaguars (*Panthera onca*) or pumas (*Puma concolor*) [28,57].

As expected, body mass was negatively correlated with occupancy, yet, this correlation was significant only in the Neotropics. The low occupancy of large mammals can be explained by a combination of intrinsic traits (e.g. low reproductive rates, high metabolic rates) and environmental or anthropogenic factors (e.g. high hunting pressure and high vulnerability to fragmentation as large species require large home ranges) [26]. In fact, previous research has shown that hunting preferences in the Neotropics are highly biased toward larger versus smaller species [28]. This conclusion is consistent with indications that the mean body mass of mammal communities in tropical forests increases with distance from human settlements and reduced human pressure [4]. Additionally, it is important to highlight that the Neotropics lost many of its largest herbivore species during the Quaternary with few now remaining (e.g. *Tapirus bairdii* or *Tapirus terrestris*) [24].

### (b) Occupancy specialization relationships differ among biogeographic regions

A key finding from our analysis was that the relationship between mammal occupancy and habitat specialization differed among biogeographic regions. Previous studies suggest that specialists are typically more affected than generalists by human activities and impacts [29,58–60]. Here, we investigated the relationship between habitat specialization and occupancy in tropical protected areas, where anthropogenic impacts are minimal. As expected, in most locations occupancy was higher for habitat specialists than for habitat generalists, especially in Afrotropical protected areas (electronic supplementary material, figure S6). Surprisingly the Indo-Malayan region was a striking exception where specialists had lowest occupancy (electronic supplementary material, figure S6). Here, we focused on protected areas with high forest cover and where habitat destruction rarely occurs. While habitat destruction can be easily monitored with remote-sensing techniques, other threats such as poaching are undetectable and thus, difficult to monitor, even inside protected areas [52]. The low occupancy of habitat specialists in the Indo-Malayan region may reflect anthropogenic pressure that these protected areas experience, especially along the protected area borders. Global data on human population density shows that the Indo-Malayan protected areas assessed in this study have a relatively high human density in their surrounding (electronic supplementary material, figure S4), which may explain why generalists in these protected areas had higher occupancy than specialists (electronic supplementary material, figure S6). Our occupancy estimates also showed that overall, all species had lower occupancy in the Indo-Malayan region than in the Afrotropical or Neotropical region (with the latter having the highest occupancy values) (figure 3). This further suggests that anthropogenic threats may have overall higher impacts on mammal communities in the Indo-Malayan region than in the Neotropics or Afrotropics. More work would be needed to clarify the implications of this difference and to see if it holds more generally, but it suggests that protected areas alone may be less effective for protection of habitat specialists in the Indo-Malayan region and that other conservation activities may be of higher priority than in other regions. These include poaching control or expanding forested areas through restoration or by increasing connectivity between forest patches. Other factors, such as variation in forest diversity appear less likely to explain differences in the occupancy-specialization relationship across regions. In the Neotropics, where local and regional plant species richness is especially high [24], this relationship was weak.

### (c) Mammal occupancy decreases with human population but increases with forest fragmentation

Overall, we found that mammal occupancy was negatively correlated with surrounding human population. This seems reasonable given that human activities such as agriculture, logging or hunting are the most persistent threats to biodiversity in the tropics [61] and is in line with other large-scale studies that assessed the effects of human disturbances on species richness [62] or functional diversity [9]. An exception was the pattern observed in the Indo-Malayan region, where occupancy increased with human population. Although this negative relationship was not significant, it contributes to explain the high occupancy of habitat generalists in the Indo-Malayan region and supports previous findings that generalists may cope with human-induced changes better than specialists [58,60].

We found that forest fragmentation was positively correlated with mammal occupancy in the Neotropics and Afrotropics. Despite substantial research efforts, the effects of habitat fragmentation on biodiversity remains inconclusive [39,63,64]. Here, we assessed how large-scale fragmentation (10 km buffer around the camera-trap arrays) correlates with mammal occupancy inside protected areas. Given that forest cover in the protected areas is high, the effects of fragmentation are most likely driven by the landscape characteristics outside the protected area boundaries. This is the case of the Udzungwa Mountains National Park in Tanzania, where forest fragmentation in the surroundings is high. Therefore, it may be that occupancy inside the protected areas increases when fragmentation increases in the surroundings, as individual are pushed to occupy protected areas where habitat availability is high, and fragmentation is low. Likely too, where species are able to move among and occupy fragments they may benefit from these conditions, though we expect marked differences among species and regions. For example, we found the strongest positive effect of fragmentation on mammal occupancy in the Afrotropics, where we know that most otherwise arboreal species readily cross open ground (for example virtually all the diurnal primates and civets [65]). By contrast, in the Indo-Malayan region, where fragmentation effects were negative (although non-significant), several species of civet, including the small-toothed palm civet (*Arctogalidia trivirgata*) and the binturong (*Arctictis binturong*), and various primates including leaf monkeys and gibbons are unwilling to move over open ground [66]. Including covariates that represent other components of habitat fragmentation (e.g. distance to edge or patch size) in the model may further clarify the effects of forest fragmentation on occupancy patterns. In future work we hope to clarify measures of habitat availability and fragmentation in influencing these results.

### (d) Detection

While patterns in detection probabilities generally followed expectations there were anomalies. Detection for ground-dwelling species was higher than for arboreal or scansorial species as expected given camera placement. Detection probability generally decreased with body mass. This is not surprising as larger species tend to have large home-range sizes and low population densities [67], making detection less likely than for smaller species that typically occur at higher densities and restrict their activities to a smaller area. An exception was observed for arboreal and scansorial species from the Afrotropical and Indo-Malayan regions, where larger species had higher detection probability than smaller species. The Afrotropics support a greater proportion of species adapted to open and fragmented forests [68] and it appears that many larger arboreal and scansorial species such as chimpanzees (*Pan troglodytes*) spend considerable time on the ground [69]. Processes operating on these detection probabilities in the Indo-Malayan region likely relate to anthropogenic activities and impacts. A study from Borneo showed that the degree to which arboreal species use the ground depends on forest structure, and large species such as orangutans spend more time on the ground with canopy disruptions [70]. Including canopy structure differences at the site level in our models might help to clarify such differences in detection probabilities.

## 5. Conclusion

We used standardized camera-trapping and hierarchical models to compare occupancy patterns for tropical forest mammals across biogeographic regions. We found that occupancy relates to feeding guild, body mass and habitat specialization and that these relationships differ among regions. These results challenge assumptions of convergence in community structure that might be explained by evolution under relatively similar environmental conditions [4,7], and underlines that the uniqueness of the forest mammal communities in each tropical region includes not just taxonomy but occupancy patterns and associated ecologies. Differences in occupancy patterns across regions were mainly explained by species' degree of habitat specialization. Our results suggest that protected forests, as might be expected, particularly benefit specialist species, often at a higher risk of extinction and the focus of conservation efforts [29,71]. This pattern is not evident in the Indo-Malayan region, where additional conservation actions appear needed to increase the occupancy of habitat specialists.

## Data Availability

Raw camera-trap data from the TEAM Network are available on the Wildlife Insights platform (wildlifeinsights.org). Our forest-cover and human population data are based on publicly available data, and extracted values are in a Dryad Digital Repository, as well as the R script to subset and organize the data (doi:10.5061/dryad.n02v6wx0n [72]). Species covariates, model code and detailed modelling results are included in the electronic supplementary material [73].
